# Pneumococcal Nasopharyngeal Carriage in Young Healthy Children After Pneumococcal Conjugate Vaccine in Turkey

**DOI:** 10.4274/balkanmedj.2016.1256

**Published:** 2017-08-04

**Authors:** Ahmet Arvas, Haluk Çokuğraş, Emel Gür, Nevriye Gönüllü, Zeynep Taner, Hrisi Bahar Tokman

**Affiliations:** 1 Department of Paediatrics, İstanbul University Cerrahpaşa School of Medicine, İstanbul, Turkey; 2 Department of Medical Microbiology, İstanbul University Cerrahpaşa School of Medicine, İstanbul, Turkey

**Keywords:** children, nasopharyngeal carriage, pneumococcal conjugate vaccine, Streptococcus pneumoniae

## Abstract

**Aims:**

To determine the prevalence of nasopharyngeal carriage of Streptococcus pneumoniae in healthy children aged 0-6 years who were vaccinated with pneumococcal conjugate vaccine.

**Methods:**

This cross-sectional study was conducted on 150 healthy Turkish children between 1 month and 6 years of age. Serotyping was performed and risk factors of carriage were evaluated.

**Results:**

The overall carriage rate was 14%. Vaccine type serotypes were determined in 17 (12.6%) children who received full-dose PCV13 vaccine. The highest carriage rate was observed among children younger than 24 months (76.2%). In multivariate analysis, respiratory infection in recent months, age, attendance at a day-care center and antibiotic usage were not statistically significant risk factors for carriage. Overall, S. pneumoniae strains were considered as penicillin susceptible and antimicrobial resistance was limited.

**Conclusion:**

We observed a low rate of pneumococcal carriage in children after PCV13 implementation compared with that of children receiving PCV7. Although it was reduced, vaccine serotype colonization in PCV13-vaccinated children remains persistent.

Vaccine serotype (VT)-dependent invasive pneumococcal diseases (IPD) have been reduced significantly in immunised children and in non-vaccinated populations in countries where pneumococcal conjugate vaccine (PCV) has been introduced into the national immunisation program (NIP). PCV vaccine also reduces nasopharyngeal (NP) pneumococcal carriage and confers herd immunity on unvaccinated children and sensitive adults (1).

Pneumococcal 7-valent vaccine (PCV7, serotype 4, 6B, 9V, 14, 18C, 19F and 23F) has been shown to be highly effective in reducing VT-dependent IPD and hospitalization. In addition, PCV7 has significantly decreased the prevalence of VT carriage (2); however, there has also been a relative increase in the frequency of infections due to non-VT (NVT) such as 19A (3). The 13-valent PCV (PCV13), which comprises six additional serotypes (1, 3, 5, 6A, 7F, 19A) in addition to the serotypes in PCV7, has been widely used in immunisation programs since 2010. The efficacy of PCV13 against IPD varies between countries (4,5).

The purpose of this study was to determine the carriage rate of NP Streptococcus pneumoniae in healthy children aged 0-6 years who were vaccinated with PCV and the serotype distribution and antimicrobial resistance of isolates.

## MATERIALS AND METHODS

This cross-sectional study involved 150 healthy Turkish children aged 0-6 years, attending the well-baby outpatient clinic in İstanbul University Cerrahpaşa School of Medicine, Department of Paediatrics between September and December 2014. PCV7 vaccine was implemented in the NIP in November 2008 as a 3+1 dose schedule at 2, 4, 6 and 12 months. It was replaced by PCV13 in April 2011, on the same schedule. The vaccination status of the children was determined as fully vaccinated (3 or 4 doses). A control group (unvaccinated children) was not included in this study, because the vaccination rate in Turkey is very high (98%). Exclusion criteria for enrolment were acute (e.g., respiratory infection) and chronic diseases. Information was obtained from parents using a questionnaire on demographics, household properties, risk factors for carriage, history of upper respiratory tract infections and antimicrobial use.

NP specimens were transferred to the microbiology laboratory in liquid Amie’s transport medium (Copan Diagnostics, Brescia, It.). The swabs were streaked on 5% sheep’s blood agar chocolate agar plates. Gram-positive diplococci appearance in gram-stained smears were assessed as streptococci. The identification of S. pneumoniae was confirmed via optochin susceptibility and bile solubility testing. The serotypes of pneumococcal isolates including PCV13 serotypes and non-PCV13 serotypes (7A, 7B, 7C, 19A, 19B, 19C) were detected using a commercial latex agglutination kit (Pneumotest-Latex kit; Statens Serum Institude, Denmark). Confirmation and factor typing were performed by the capsular reaction test (Quellung reaction) using specific antisera (SSI). Antimicrobial susceptibility testing of S. pneumoniae strains was performed according to standard procedures as previously described. The minimum inhibitory concentration values of antibiotics were determined via the E test (Etest, Biomerieux, Paris, France). The susceptibility results were evaluated according to the European Committee on Antimicrobial Susceptibility Testing (EUCAST 2015) guidelines (6,7,8).

The study was approved by the Ethics Committee of İstanbul University Cerrahpaşa School of Medicine (A-50). Written informed consent was provided by the parents of each enrolled child. This study was funded by the Turkish Paediatric Association (grant number: 05).

### Statistical analysis

The software program SPSS 22.0 was used for statistical analyses. Frequencies for categorical variables were evaluated by percentiles. Continuous variables, if they fit with the normal distribution were expressed as means ± standard deviation; if they did not fit, they were expressed as medians (minimum-maximum). The relationship between NP carriage and age was evaluated by chi-square test. The independent variables affecting NP carriage were identified by univariate analysis and were evaluated with odds ratios and 95% confidence intervals (CI). Significant variables therein were subjected to multivariate logistic regression analysis.

## RESULTS

The mean age of the children was 23.7±14.3 months; 49% of the participants were males. More than 90% of the children had received full doses of PCV13 (3 or 3+1). Descriptive characteristics of the children are presented in Table 1.

The overall NP pneumococcal carriage rate was 14% (21/150). The NP carriage rate of children vaccinated with PCV7 (3+1) was 21.4% (3/14), whereas it was 11.7% (6/51) in children vaccinated with PCV13 (3 doses) and 14.4% (12/83 children) in those with PCV13 (3+1). The highest carriage rate of 76.2% (16/21 children) was observed among children younger than 24 months with no significant differences between age groups (p=0.495) (Table 2).

Twenty carriers had more than one serotype. Of the 81 isolates, nine different serotypes were identified. Thirty-six isolates were PCV13 VTs, whereas 44 isolates were non-VT isolates. Three isolates had serotypes included in PCV7, whereas nine isolates did not. 

The risk factors of having siblings, people living at home, day care attendance and smoking in the home were found not to affect NP carriage (p=0.572, p=0.900, p=0.145 and p=0.406 respectively), whereas NP carriage was associated with a respiratory infection in the last 3 months (p=0.048). Univariate and multivariate logistic regression analysis results are shown in Table 3. We did not detect any risk factors in the multivariate analysis.

**Figure 1:**
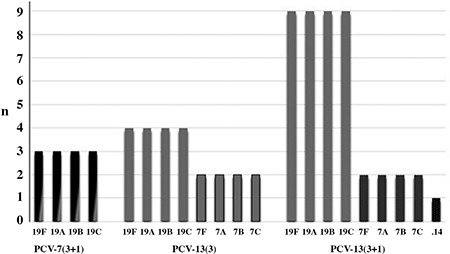
Serotype distribution of the nasopharyngeal carriage (n=21) according to the vaccination status PCV: pneumococcal conjugate vaccine

Serotypes 19A, 19B, 19C and 19F were detected in three children vaccinated with PCV7 (3+1 doses). Serotypes 7A, 7B, 7C and 7F were detected in two children vaccinated with PCV13 (3 doses), whereas serotypes 19A, 19B, 19C and 19F were detected in four children vaccinated with the same doses. Serotypes 7A, 7B, 7C and 7F were detected in two children vaccinated with PCV13 (3+1 doses), whereas serotypes 19A, 19B, 19C and 19F were in nine children vaccinated with the same doses (Figure 1). 

Penicillin susceptibility was identified in 85.7% of isolated strains. All isolates (100%) were vancomycin susceptible, whereas 85.7% of them were susceptible to cefaclore, 52.3% were susceptible to erythromycin, 47.6% were susceptible to clindamycin and 42.8% were susceptible to trimethoprim/sulphamethoxazole.

## DISCUSSION

In this study, the overall rate of NP carriage was 14% among full-dose vaccinated children. NP pneumococcal colonization varies according to region and community. In developing countries, the rate of NP carriage in children under 5 years of age was found to be 8%-75%, and in developed countries, it was 13%-77% before the introduction of PCV into NP (9). Several surveys have documented the effect of PCV on NP carriage with conflicting results (10,11,12). The main reason for the unchanged prevalence of NP carriage is serotype replacement of non-vaccine types (13,14). After the introduction of PCV7, the incidence of pneumococcal disease and NP carriage due to NVTs, most notably for serotype 19A, increased incrementally.

Dunais et al. (15). observed that the rate of NP carriage decreased significantly after PCV7 introduction; on the other hand, a further significant decrease was not seen following the implementation of PCV13, and the seroprevalence of 19A in NP colonization was persistent. Lee et al. (16) reported stable colonization rates after PCV13, with significant increases in non-PCV13 serotypes.

In this study, NP carriage prevalence was found to be higher in children attending a day-care centre. In a study carried out in Hungary, the NP carriage rate among unvaccinated children attending a day-care centre was also found to be higher (13). 

Özdemir et al. (17) reported that the NP carriage rate in healthy children was not influenced by PCV7 (21.9%). We suggest that the reason for the lower NP carriage rate in our study compared with the study mentioned above is the that PCV13 includes more serotypes than PCV7. Soysal et al. (18) reported 8%, 7% and 5% NP carriage rates among age groups of 0-24 months, 25-60 months and >60 months, respectively. Sixty percent of children in this study were not vaccinated with any PCV, whereas 30% of children received the full schedule (4 doses) of either PCV7 or PCV13.

We did not observe any association between NP carriage and gender, whereas some studies have reported that the male gender is a risk factor (15). The NP carriage rate in our study was higher in children who had a respiratory tract infection, similar to several other studies (11,17). We could not observe any positive effects of using antibiotics on carriage. A study in Italy reported that the use of antibiotics in the previous 3 months was associated with lower NP carriage prevalence, whereas having siblings, attending a day-care centre and having had a respiratory tract infection in the last 3 months were associated with higher NP carriage prevalence (12).

In the present study, the NP carriage rate in children vaccinated with PCV7 (3 or 3+1 doses) was found to be higher than that in children vaccinated with PCV13 (18.7% vs 13.4%). The reason for more effective prevention by PCV13 than PCV7 is that it contains more serotypes, similar to the findings of other studies (12,16,19).

We observed that 3 children vaccinated with PCV7 had the 19F serotype, 4 children vaccinated with PCV13 (3/3+1 doses) had 7F and 13 cases vaccinated with PCV13 had 19A and 19F serotypes. A study by Grivea et al. (19) determined that 15.8% of the children attending a day-care centre had the 19A serotype 6 months after PCV13 vaccination. In addition, the authors reported that, although there was no 19F serotype carriage in the first year of PCV7 vaccination, after the first year, the prevalence of serotype 19F was 2.2%. NP colonization containing VTs is also noteworthy in some studies, and this situation can be explained with “immune-hyporesponsiveness to the colonizing serotype following PCV” (16,20).

In our study, pneumococcal isolates in NP colonization were 85% penicillin sensitive. In a study by Zuccotti et al. (12) in Italy, it was observed that 69% of the pneumococcus isolates from NP carriers after PCV-13 vaccination were penicillin sensitive, 29% were intermediate sensitive and the most frequently isolated resistant serotype was 19F.

Studies similar to ours reported that, following PCV introduction, a significant reduction was observed in penicillin-resistant colonizing S. pneumoniae isolates due to VTs. Several studies observed that the 19A serotype was the most resistant (10,13,15).

There are some limitations to this study. First, we had no pre-PCV period cohort with which to compare our study population. Second, this study is a cross-sectional study. Due to the lack of a long-term surveillance study of the same cases, it will not be possible to determine whether the NP carriage frequency and serotypes change in the future. Third, the present study has a relatively small sample size and does not include children older than 6 years. Therefore, it is not possible to determine the frequency of NP pneumococcal carriage in all children.

In conclusion, this study determined the lower NP carriage prevalence after PCV-13 implementation compared with previous studies before or after PCV-7 implementation. This study also determined that, in the early childhood years, only having a respiratory tract infection has a positive effect on NP colonization. There are some VTs of NP carriage with low frequency. In Turkey, where the coverage rate of vaccination in children is quite high, studies including a large sample size and a broader age range would be more useful in determining the actual long-term NP carriage status, which is very important in IPD and herd immunity.

## Figures and Tables

**Table 1:**
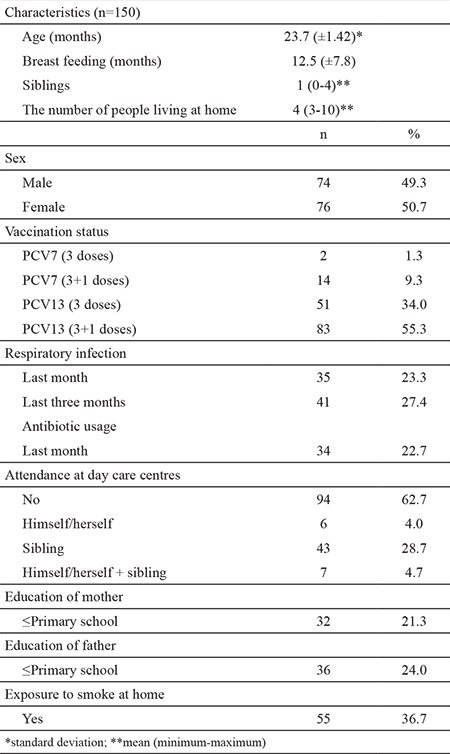
Characteristics of the study population

**Table 2:**
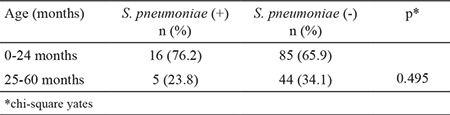
Relationship between pneumococcal carriage and age in children

**Table 3:**
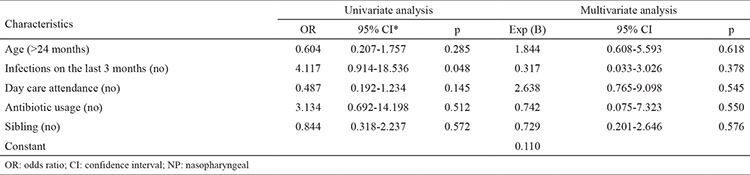
Examination of the risk cases for NP carriage in univariate and multivariate analyses
